# Supplementary material for: Preoperative MRI and LDH in women undergoing intra-abdominal surgery for fibroids: Effect on surgical route
Source: PLoS One. 2021 Feb 9;16(2):e0246807. doi: 10.1371/journal.pone.0246807 (PMC7872248; doi:10.1371/journal.pone.0246807)
Supplement: S1 Table — (DOCX) [file pone.0246807.s001.docx]

S1 Table: Surgical route stratified by MRI/LDH results in post-protocol cases where both MRI and LDH test was done according to the protocol.

| **Test** | **Result** | **Total (N)** | **Open procedure**  **(N, %)** | **P-value**^*^ |
| --- | --- | --- | --- | --- |
| **MRI** | Normal | 238 | 8; 3.4% | **0.001** |
| **MRI** | Abnormal | 120 | 16; 13.3% |  |
| **LDH** | Normal | 282 | 16; 5.7% | 0.19 |
| **LDH** | Abnormal | 76 | 8; 10.5% |  |
| **MRI/LDH** | Both normal | 203 | 6; 3.0% | Reference |
| **MRI/LDH** | Both abnormal | 23 | 4; 17.4% | **0.01** |
| **MRI/LDH** | Normal/abnormal | 35 | 2; 5.7% | 0.33 |
| **MRI/LDH** | Abnormal/normal | 97 | 9; 9.3% | **0.02** |

MRI-Magnetic Resonance Imaging

LDH- Lactate Dehydrogenase enzyme

^*^Corresponds to Fisher’s Exact test of association between reference group and other combinations of MRI and LDH results.
