# Supplementary material for: Preoperative MRI and LDH in women undergoing intra-abdominal surgery for fibroids: Effect on surgical route
Source: PLoS One. 2021 Feb 9;16(2):e0246807. doi: 10.1371/journal.pone.0246807 (PMC7872248; doi:10.1371/journal.pone.0246807)
Supplement: Attachment — Submitted filename: response to reviewers.docx [file pone.0246807.s003.docx]

PONE-D-20-37531
Preoperative MRI and LDH in women undergoing intra-abdominal surgery for fibroids: effect on surgical route.

Dear Editor:

Thank you for the thorough review of our manuscript and the insightful and useful comments and queries. We have incorporated the feedback and are resubmitting the manuscript for your consideration. Below we respond point-by point to the reviewer comments as you have requested. We appreciate the feedback and believe the paper is stronger as a result of your suggestions and questions.

We look forward to hearing back from you,

Veronica Lerner, MD

| **Editor and Reviewer Comments** | **Author’s response indicating (using line numbers) where/how the manuscript has been revised to address the comment (if relevant).** |
| --- | --- |
| Please complete your Competing Interests on the online submission form to state any Competing Interests. If you have no competing interests, please state "The authors have declared that no competing interests exist.", as detailed online in our guide for authors at [http://journals.plos.org/plosone/s/submit-now](about:blank) | The authors have declared that no competing interests exist |
| At this time, please address the following queries:     a. Please clarify the sources of funding (financial or material support) for your study. List the grants or organizations that supported your study, including funding received from your institution. b. State what role the funders took in the study. If the funders had no role in your study, please state: “The funders had no role in study design, data collection and analysis, decision to publish, or preparation of the manuscript.” c. If any authors received a salary from any of your funders, please state which authors and which funders. d. If you did not receive any funding for this study, please state: “The authors received no specific funding for this work.”             Please include your amended statements within your cover letter; we will change the online submission form on your behalf. | 1. no funders or funding 2. n/a-no funders 3. no salary as no funders 4. “The authors received no specific funding for this work.” |
| **Reviewer #1** |  |
| Reviewer #1: I read with great interest the manuscript, which falls within the aim of this Journal. In my honest opinion, the topic is interesting enough to attract the readers’ attention.  Nevertheless, authors should clarify some points and improve the discussion, as suggested below. Authors should consider the following recommendations: | Thank you very much! |
| Accumulating evidence suggests that some intrinsic abnormalities of the myometrium, abnormal myometrial receptors for estrogen, and hormonal changes or altered responses to ischemic damage during the menstrual period may be responsible for the initiation of (epi)genetic changes found in uterine myomas. I would stress this point of paramount importance, referring to: PMID: 28875276 | Thank you for your suggestion.  The following sentence has been added to discussion incorporating suggested reference PMID: 28875276:  “Finally, as scientific advancements are made in understanding epigenetic and genetic landscape of uterine myomas, we hope that our treatment options will expand beyond what is available as this time.[18]” |
| - To date, the pre-operative diagnosis and post-operative management of uterine sarcomas represent still a challenge. I truly think that it would be necessary to add few lines about the risk of facing off of a misdiagnosed sarcoma during the management of uterine myomas and how to proceed (authors may refer to: PMID: 32497607; PMID: 33098396) | The following sentence has been added to the introduction incorporating suggested references”  “Preoperative diagnosis of cancer in women presenting with abnormal uterine bleeding remains a challenge.[6, 7]” |
| **Reviewer #2** |  |
| Reviewer #2: the authors report a single center experience on morcellation after adopting an internal protocol for suspicious myomas. The paper is well written and clearly expose the problem. However, there are some limitations that needs to be clarified: |  |
| Line 150 -162:  please report the indication for hysterectomy for patients included in the study,  has polyps been considered an indication for hysterectomy?  Would have been useful to include only hysterectomies for fibroids | The following was added  “We included women between the ages of 18-99 with symptomatic fibroid uterus who underwent intra-abdominal surgery for leiomyomas. This included myomectomies (laparoscopic, robotic and open approaches) and hysterectomies (both total and supracervical, with laparoscopic, robotic, vaginal, laparoscopically-assisted vaginal and open approaches). We excluded hysteroscopic myomectomies. Patients were screened by a review of the operating room schedule during the study period. Eligible patients’ charts were reviewed to extract relevant data including: patient demographics, preoperative workup, type of surgery and method of tissue extraction. We reviewed our internal institutional gynecologic cancer database (created and maintained for the purposes of tumor board treatment planning) to confirm that all cases of LMS were captured in our study. ”  Polyps are not considered to be an indication. Would you like us to add that to the paper?  Sorry not able to follow. Please explain. |
| Line 178- the authors report a protocol compliance of 59% that actually create a bias in interpreting the results. | The following sentence was modified to emphasize risk of election bias  “Future work could be done to address the relatively low rate of compliance with this protocol, which could lead to concerns with selection bias.” |
| Line 179 - 191: The data reported here are actually duplicated in table number 6 that is difficult to be read, i suggest to remove the table | Table 6 moved to supplement |
| The pre and post protocol analysis has a limited validity because of the too low compliance to the protocol therefore this should be considered a snapshot of the surgeon's decisional process following the 2014 facts.  Would be nice to see if there is trend in the last 6 years and how the patients that did no underwent MRI were treated. | We plan to conduct a follow up study, but for this paper it is beyond the scope of the project. |
| The fact that only 59% of the patients underwent both MRI and LDH means that in the centre there are other parameters used to stratify the risk, please report the how the patients were selected for the screening. | No parameters are used. Each surgeon decides on their own.  The following sentence has been added  “While only 59% of patients have undergone both MRI and LDH testing, our hospital did not have any other parameters for patient selection, and in the future, it might be of interest to study how each individual surgeon made decisions at the time of pre-operative work up.” |
| The paper would benefit of a post operative care analysis such as Hb drop, hospitalization etc to understand the real impact of the morcellation issue on our patients health. | This question was not in study aims. It has been extensively reported on in the past. |
